# Polygenic Risk Score, Lifestyles, and Type 2 Diabetes Risk: A Prospective Chinese Cohort Study

**DOI:** 10.3390/nu15092144

**Published:** 2023-04-29

**Authors:** Jia Liu, Lu Wang, Xuan Cui, Qian Shen, Dun Wu, Man Yang, Yunqiu Dong, Yongchao Liu, Hai Chen, Zhijie Yang, Yaqi Liu, Meng Zhu, Hongxia Ma, Guangfu Jin, Yun Qian

**Affiliations:** 1 Department of Chronic Non-Communicable Disease Control, The Affiliated Wuxi Center for Disease Control and Prevention of Nanjing Medical University (Wuxi Center for Disease Control and Prevention), Wuxi 214023, China; 2 Department of Epidemiology and Biostatistics, School of Public Health, Nanjing Medical University, Nanjing 211166, China; 3 College of Arts and Science, University of North Carolina, Chapel Hill, NC 27599, USA

**Keywords:** polygenic risk score, lifestyle, cohort, type 2 diabetes

## Abstract

The aim of this study was to generate a polygenic risk score (PRS) for type 2 diabetes (T2D) and test whether it could be used in identifying high-risk individuals for lifestyle intervention in a Chinese cohort. We genotyped 80 genetic variants among 5024 participants without non-communicable diseases at baseline in the Wuxi Non-Communicable Diseases cohort (Wuxi NCDs cohort). During the follow-up period of 14 years, 440 cases of T2D were newly diagnosed. Using Cox regression, we found that the PRS of 46 SNPs identified by the East Asians was relevant to the future T2D. Participants with a high PRS (top quintile) had a two-fold higher risk of T2D than the bottom quintile (hazard ratio: 2.06, 95% confidence interval: 1.42–2.97). Lifestyle factors were considered, including cigarette smoking, alcohol consumption, physical exercise, diet, body mass index (BMI), and waist circumference (WC). Among high-PRS individuals, the 10-year incidence of T2D slumped from 6.77% to 3.28% for participants having ideal lifestyles (4–6 healthy lifestyle factors) compared with poor lifestyles (0-2 healthy lifestyle factors). When integrating the high PRS, the 10-year T2D risk of low-clinical-risk individuals exceeded that of high-clinical-risk individuals with a low PRS (3.34% vs. 2.91%). These findings suggest that the PRS of 46 SNPs could be used in identifying high-risk individuals and improve the risk stratification defined by traditional clinical risk factors for T2D. Healthy lifestyles can reduce the risk of a high PRS, which indicates the potential utility in early screening and precise prevention.

## 1. Introduction

Type 2 diabetes (T2D) has become an urgent public health problem worldwide. Globally, age-standardized mortality rates for diabetes show an adverse trend, increasing by 3% from 2000 to 2019 [1]. According to the report of the International Diabetes Federation (IDF), 537 million adults are living with diabetes and 6.7 million deaths of diabetes occurred worldwide in 2021; more than one in four new cases of and one in five deaths from diabetes occurred in China [2]. Genetic predisposition, unhealthy lifestyles, and an aging population could lead to a rapid increase in the burden of diabetes among the Chinese [3]. Traditional risk factors, including unhealthy dietary patterns, physical inactivity, and general and central obesity, have been shown to be associated with an increased risk of T2D [4,5]. Intensive intervention, including modifying diet, losing weight, and regular physical exercise, can effectively prevent or delay T2D occurrence [6,7,8], indicating that lifestyle intervention can be regarded as a potential feasible way to prevent T2D. However, resource constraints make it impossible to promote intensive intervention in the whole population. Identifying high-risk individuals and implementing a precise intervention may improve the effectiveness of T2D prevention and control.

The heritability of T2D has been estimated to be 41% in the Chinese population from Chinese adult twins [9], while it is 26–78% in the European population [10,11]. An increased genetic risk could cause early onset of the disease [12]. To date, many genetic variants for T2D risk have been identified by genome-wide association studies (GWAS). Studies have shown that polygenic risk scores (PRSs), which aggregate multiple common loci with low impact into risk models, can effectively predict chronic non-communicable diseases (NCDs), such as cancer, stroke, and coronary artery disease, providing an opportunity for clinical application [13,14,15,16,17]. The high genetic risk of NCDs (including coronary artery disease, stroke, and cancer) could be counteracted by adopting a healthy lifestyle [14,18,19]. However, no standard analysis was conducted to evaluate whether high-genetic-risk individuals could be identified by the T2D PRS for potential personalized, precise prevention in Chinese Hans. In addition, the joint effect of genetic risk and lifestyle remains unclear. Furthermore, the Chinese diabetes risk score (CDRS) (which consists of six traditional clinical risk factors: age, sex, systolic blood pressure, body mass index, waist circumference, and diabetes family history) is recommended to identify high-risk individuals for intensive intervention by the Chinese Guidelines for Diabetes Prevention and Treatment (2020 edition) [20,21,22]. Individuals with a score of ≥25 are regarded as at high risk. However, it remains unclear whether adding PRS could optimize the risk stratification of T2D beyond traditional risk factors.

In this current study, we generated a PRS for T2D and tested its utility and effectiveness in predicting T2D in a prospective cohort of Chinese with 14 years of follow-up. Furthermore, we assessed the public health significance of the PRS in identifying high-genetic-risk individuals for a healthy lifestyle intervention to prevent T2D incidents. Then, we evaluated whether the PRS could refine the risk stratification defined by the traditional clinical risk score for T2D to identify the high-risk population effectively.

## 2. Materials and Methods

### 2.1. Study Design and Participants

The Wuxi Non-Communicable Diseases cohort (Wuxi NCDs cohort) was established from April to June 2007 (baseline) in Wuxi city, Jiangsu Province, China. A total of 10,858 adults aged ≥30 years old were recruited with random cluster sampling from 2 communities. As previously described [23], all participants completed a face-to-face questionnaire, underwent physical measurements, and provided peripheral venous blood samples for further examinations and DNA extraction. The questionnaire included general population sociological characteristics, lifestyle, and disease history. The physical measurements, including height, weight, waist circumference (WC), and blood pressure, were measured using standard instruments and protocols. The height and weight were measured for the participants wearing light clothes using a digital weight and height scale. Body mass index (BMI) was calculated as weight (kg) divided by height square (m^2^). Waist circumference was measured with a tape around about 5 cm above the umbilicus for each participant at a relaxed state. Each participant was measured twice and the average of the two measurements was calculated. Then, 5% of the participants were randomly selected for quality control surveys, repeated questionnaires, and physical measurements. Each participant donated 5 mL of fasting venous blood. Several indices, including fasting blood glucose (FBG), triglyceride (TG), total cholesterol (TC), and high-density lipoprotein cholesterol (HDL-C), were measured by a Biochemistry Auto-analyzer.

With the cohort study design, first we examined the association between healthy lifestyle, genetic risk, and onset of T2D risk during the follow-up period. Then, we evaluated the combined effect of lifestyle and genetic risk categories on T2D risk across different genetic risk groups. Third, the benefit of adhering to a healthy lifestyle was assessed between different genetic risk groups. Last, we evaluated whether adding PRS could refine risk stratification defined by the current guideline-recommended risk score defined by established risk factors. 

### 2.2. Outcome Ascertainment

Annual follow-up was conducted primarily through linkages with existing national and local data sets. Mortality data were obtained from the China National Disease Surveillance Points (DSP) system, which was published by the Chinese CDC. The DPS system collects information on medical certificates for all deaths [24]. Information about incident T2D was collected through the local chronic non-communicable disease (NCD) registration system, health record management system, and hospital information system established by Wuxi Health Commission, Wuxi CDC, and the Wuxi Center for Health Statistics and Information. Once the participants were diagnosed with T2D, the demographics and ICD-coded diagnoses information could be obtained from the NCD registration system and the hospital information system. Medical records are reviewed by trained and qualified staff and necessary clinical information is collected for diagnosis verification. The outcome, in the final analysis, was type 2 diabetes (ICD-10 code: E11.X). 

By June 2021, a total of 9796 participants (follow-up rate 90.22%) had completed follow-up. We excluded 4328 participants with diabetes (self-reported diabetes history or FBG ≥ 7.0 mmol/L), hypertension (self-reported hypertension history), cancer, and cardiovascular and cerebrovascular disease at baseline, and 444 participants due to the poor DNA quality for genotyping assay (Figure S1). Finally, 5024 participants were examined for the effect of PRS and traditional risk factors on incident T2D.

After baseline recruitment, T2D risk was evaluated from baseline setup to T2D diagnosis, death, or completion of follow-up (June 2021), whichever occurred first. For participants who developed T2D, time was calculated by the date of T2D diagnosis minus the date of baseline setup. For participants who died during the period of follow-up without T2D, time was the interval between the date of death and the date of baseline setup. For the other participants, time at risk was calculated by the end date of follow-up minus the date of baseline.

### 2.3. Definition of the Lifestyle Score

Lifestyle factors were considered, including cigarette smoking, alcohol consumption, physical exercise, diet, body mass index (BMI), and waist circumference (WC). The six factors have also been highlighted in recent studies and guidelines for the prevention and control of T2D [25,26,27,28].

In this current study, participants were defined as smokers if they had smoked an average of one cigarette or more per day and for at least half a year in their lifetime [27]. Favorable smoking habit was defined as never smoked. Alcohol drinking was defined as consuming alcohol at least once per week and for more than half a year [27]. Favorable drinking habit was classified as never drank. Physical exercise was assessed as exercising for more than 20 min per time and for at least 3 times per week [29]. For dietary factors, a healthy diet was defined as a light diet with restriction of sodium intake and eating fried or smoked food less than 3 times per week [30]. For general adiposity measured by BMI, based on the standard classification for the Chinese population [31], overweight and obesity were defined as BMIs of 24–27.9 and ≥28 kg/m^2^, respectively. Central adiposity was defined as those who had a WC ≥ 90 cm for men and ≥85 cm for women [32]. A healthy lifestyle was defined as non-smoking, non-drinking, physical exercise, healthy diet, and normal BMI and WC. We gave one point for each favorable lifestyle factor and zero points for unfavorable lifestyle factors. We summed the score generated from each lifestyle to build a lifestyle score. The lifestyle score ranged from 0 to 6, with a higher number indicating a healthier lifestyle. Overall lifestyle was categorized as poor (0–2 healthy lifestyle factors), intermediate (3 healthy lifestyle factors), and ideal (4–6 healthy lifestyle factors). This healthy lifestyle score was established in a Dongfeng-Tongji cohort. Compared with poor lifestyle, participants with intermediate and ideal lifestyle had a 27% and 51% lower risk of T2D, respectively. We added this in the current revised version [27].

### 2.4. PRS Construction

We systemically searched and summarized genetic loci from a published T2D GWAS analysis [33,34,35,36,37,38,39,40,41,42,43,44,45,46,47,48,49,50,51,52,53,54,55,56]. Preliminarily, 114 single nucleotide polymorphisms (SNPs) were selected. We selected the SNPs based on the following criteria: (1) derived European or Eastern Asian ancestry; (2) Located at autosome; (3) minor allele frequency (MAF) > 0.05; (4) low linkage disequilibrium with others (*r*^2^ < 0.8); (5) call rate > 95%. The flow chart of the selection process of SNPs is summarized in Figure S1. Ultimately, 80 SNPs were included. Information about SNPs is presented in Table S1.

Genomic DNA was extracted from leukocytes by the method of phenol chloroform extraction, followed by ethanol precipitation. For the participants, SNPs were genotyped by the Iplex Sequenom MassARRAY platform (Sequenom, Inc., San Diego, CA, USA). For quality control, there were two non-template controls in each plate. The call rates were all >95%, except for rs7224685.

In this study, the effects of the selected SNPs were derived from the GWAS with the largest published sample size. SNPs were coded 0, 1, and 2 as the number of risk alleles. As the ethnic-specific PRS was constructed with the SNPs identified in the corresponding race [57], we constructed two PRSs based on the genotype dosage of risk allele and its weight (lnOR) reported in European or Eastern Asian population, respectively. PRS was generated by multiplying the genotype dosage of each risk allele for each variant by its respective weight (lnOR), summing all variants together [15]. It was calculated as follows:

$$\mathrm{PRS}=\sum_{i=1}^{n}\ln\left(ORi\right)\times SNPi$$

### 2.5. Statistical Analysis

The Cox proportional hazard model was used to assess associations between lifestyle, PRS, and T2D incidence, as well as a dose–response relationship (*P*_trend_), and to estimate hazard ratios (HRs) and 95% CIs with the adjustment of age, sex, blood pressure level, lipid level, fasting blood glucose, and diabetes family history with the follow-up time as the timeline variable. We assessed the proportional hazards assumptions using Schoenfeld residuals. A log-rank test was performed to compare the difference between different groups for T2D incidence probability. The PRS was categorized into low (bottom quintile), intermediate (2nd–4th quintile), and high (top quintile) genetic risk, as described [14,15]. We also compared the effect of ideal lifestyle (4–6 healthy lifestyle factors) or intermediate lifestyle (3 healthy lifestyle factors) on the risk of developing T2D with the effect of poor lifestyle (0–2 healthy lifestyle factors) using the Cox proportional hazard model. Furthermore, the joint effect of lifestyle factors and genetic factors was assessed according to lifestyle and genetic risk categories. We divided participants into 9 groups according to PRS group (low risk, intermediate risk, and high risk) and lifestyle group (ideal, intermediate, and poor), and evaluated the risk compared with the participants with ideal lifestyle and low genetic risk as reference.

Absolute risk was calculated as the percentage of T2D new cases occurring in a given population over a 10-year period. We calculated the absolute risk reduction as the difference in the incidence of T2D among given groups and extrapolated the difference in 10-year T2D events between these groups. We also calculated the number of participants required to adhere to an ideal lifestyle to prevent one case of T2D over 10 years. The 95% CIs for the absolute risk reduction was calculated by drawing 1000 bootstrap samples from the estimation data set. Cox regression was used to calculate 10-year absolute risk based on clinical risk and PRS categories. All statistical analyses were performed using R software (version 3.5.1; The R Foundation for Statistical Computing, http://www.cran.r-project.org/ (accessed on 15 April 2023)). The R packages “survival”, “forestplot”, and “bootstrap” were used. 

## 3. Results

### 3.1. Baseline Characteristics and T2D Risk

Among the 5024 participants without diabetes, hypertension, cancer, and cardiovascular and cerebrovascular disease at baseline, there were 440 T2D incidents during 14 years of follow-up. As shown in Table 1, older age, overweight/obesity, central adiposity, elevated blood pressure, elevated fasting blood glucose, and dyslipidemia were significantly associated with future T2D risk (HR ranged from 1.50 to 3.97). As for the six lifestyle factors, 26.46% and 47.90% of the participants adopted intermediate and ideal healthy lifestyles at baseline, respectively. There was a significant association between the reduction in healthy lifestyle factors and the increased risk of T2D (*P*_trend_ = 1.57 × 10^−6^). Compared with those with an ideal healthy lifestyle, the HRs of T2D in those with an intermediate and a poor lifestyle were 1.58 (95% CI = 1.23–2.03, *p* = 3.11 × 10^−4^) and 1.92 (95% CI = 1.49–2.46, *p* = 3.49 × 10^−7^), respectively. 

### 3.2. PRS and T2D Risk

**Figure 1:**
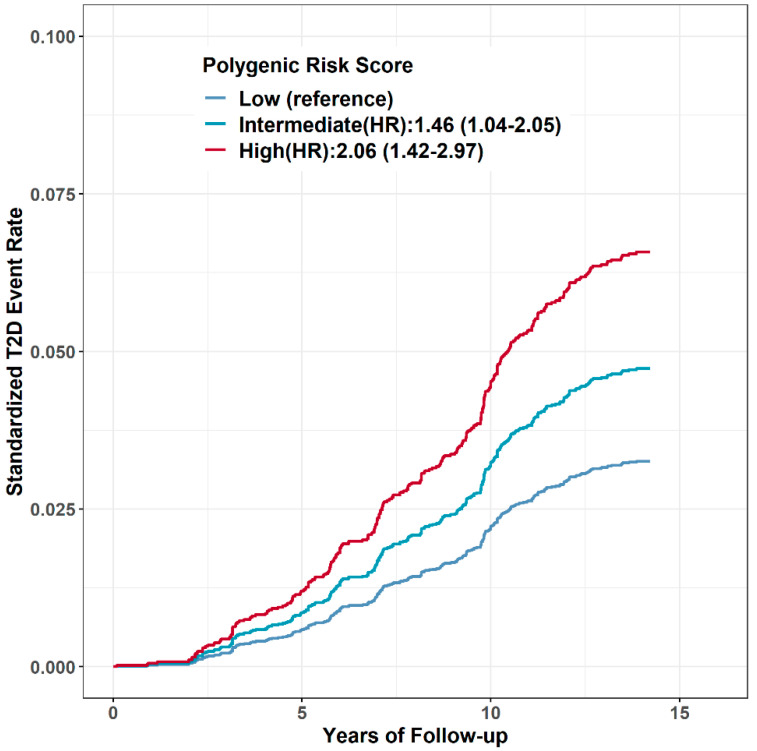
Eastern Asian PRS and the risk of incident type 2 diabetes.

Firstly, we conducted the analysis based on the Eastern Asian PRS, which was constructed by 46 SNPs identified in those with Eastern Asian ancestry (Table S1). The Eastern Asian PRS was significantly associated with incident T2D risk (HR = 1.21, 95% CI = 1.08–1.35, *p* = 6.00 × 10^−4^ per SD of PRS increase). After adjusting for baseline characteristics (age, gender, SBP, DBP, FBG, TC, TG, HDLC, and diabetes family history), compared with low-genetic-risk individuals (bottom quintile of PRS), intermediate- (second to fourth quintile) and high-genetic-risk individuals (top quintile of PRS) had a significantly higher risk of T2D, with HRs being 1.46 (95% CI = 1.04–2.05, *p* = 0.027) and 2.06 (95% CI = 1.42–2.97, *p* = 1.30 × 10^−4^), respectively (Figure 1).

Furthermore, we conducted the same analysis based on the European PRS constructed by 50 SNPs identified with European ancestry (Table S1). Compared with the low-genetic-risk group, no significant association was observed in the intermediate-genetic-risk group (HR = 1.31, 95% CI = 0.94–1.82, *p* = 0.107), while a significant association was observed in the high-genetic-risk group (HR = 1.69, 95% CI = 1.17–2.44, *p* = 0.005).

Finally, we adopted the Eastern Asian PRS for further analysis.

### 3.3. The Joint Effect of PRS and Lifestyle on T2D Risk

**Figure 2:**
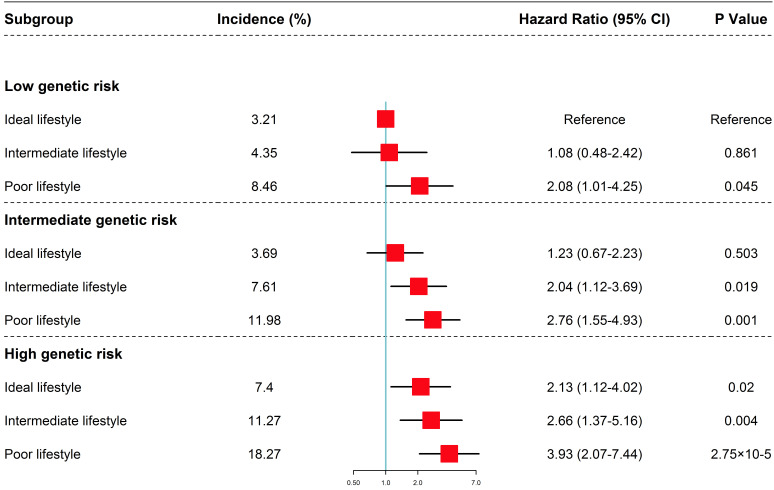
Incidence rate and risk of type 2 diabetes in different genetic risk and lifestyle groups in Wuxi NCDs cohort during 14-year follow-up.

The combined effect of PRS and lifestyle factors on the risk of T2D incidence has a dose–response relationship. The overall risk of T2D increased with the increase in PRS and poor lifestyle factors. During the 14-year follow-up, the incidence rate of T2D was rising from 3.21% in those with low genetic risk and ideal lifestyle to 18.27% in those with high genetic risk and poor lifestyle. Compared with those with low genetic risk and an ideal lifestyle, an almost four times higher risk was observed in participants with high genetic risk and poor lifestyle (HR = 3.93, 95% CI = 2.07–7.44) (Figure 2).

### 3.4. Benefits of Adhering to Ideal Lifestyle against T2D

We further conducted a stratified analysis according to different genetic risks (low, intermediate, and high). Among individuals with intermediate and high genetic risk, ideal lifestyle significantly reduced the risk of T2D, with HRs being 0.45 (95% CI: 0.30–0.68, *p* = 1.05 × 10^−4^) and 0.48 (95% CI: 0.28–0.85, *p* = 0.012), using poor lifestyle as the reference (Table 2). Among participants with high genetic risk, the standardized 10-year incident T2D rate was 6.77% for those with a poor lifestyle versus 3.28% for those with an ideal lifestyle. Similarly, among participants at intermediate genetic risk, the standardized 10-year incident T2D rate declined from 4.73% with a poor lifestyle to 2.15% with an ideal lifestyle. In order to prevent one T2D incident in 10 years, 39 participants with intermediate genetic risk should be intensively intervened to adopt an ideal lifestyle, while only 29 participants with high genetic risk should be intensively intervened.

### 3.5. Predicting T2D Risk by PRS and Traditional Clinical Risk Score

**Figure 3:**
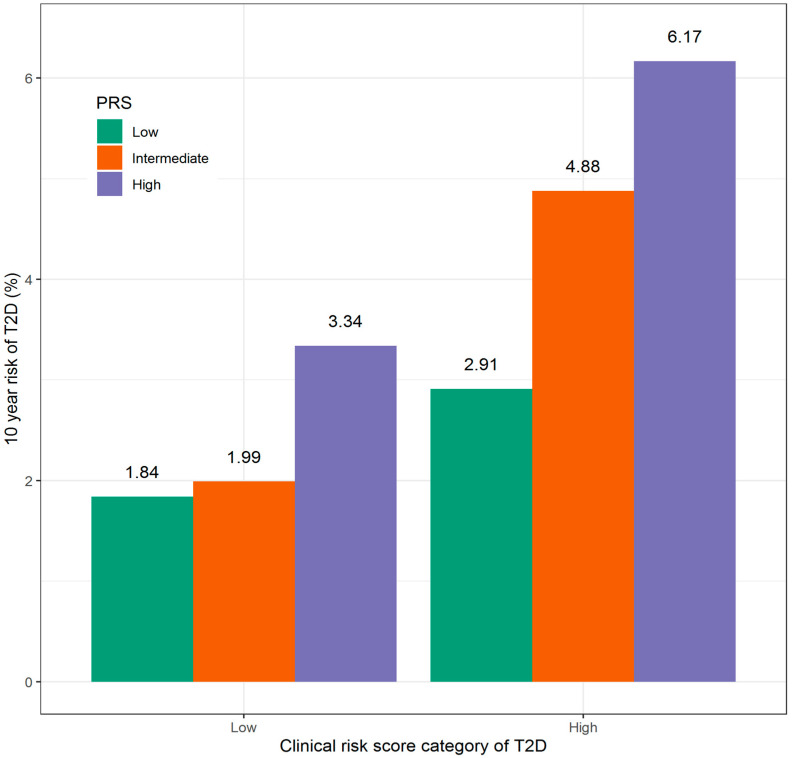
Ten-year risk of type 2 diabetes in different clinical risk scores and genetic risk groups.

According to the Chinese diabetes risk score (CDRS) recommended by the Chinese Guidelines for Diabetes Prevention and Treatment (2020 edition), 51.31% of the participants were classified into the high-risk group (CDRS ≥ 25). The CDRS demonstrated a good performance in predicting T2D incidents (HR = 1.09, 95% CI = 1.08–1.11, *p* < 2.00 × 10^−16^). Compared with the low-risk group, individuals with high clinical risk had a 2.50-fold increase in T2D risk (HR = 3.50, 95% CI = 2.80–4.38, *p* < 2.00 × 10^−16^). Interestingly, individuals with low clinical risk and high genetic risk demonstrated a 10-year T2D risk of 3.34%, which exceeded the risk levels of those in the high-clinical-risk group with low genetic risk (2.91%) (Figure 3). The hazard ratios of different groups categorized by traditional clinical risk factors and PRS are displayed in Figure S2. 

## 4. Discussion

In the current study, we generated European-derived and Eastern-Asian-derived polygenic risk scores to predict the risk of T2D in the Chinese population. The results indicated that Eastern Asian PRS could identify high-risk individuals of T2D in the Chinese Hans population, independent of traditional risk factors. Participants with high Eastern Asian PRS and poor lifestyle had a 3-fold higher risk of T2D than those with low Eastern Asian PRS and ideal lifestyle. The ideal lifestyle can reduce the risk of T2D by more than 50% in those with intermediate and high genetic risk. In order to prevent one T2D case within 10 years, 39 people with intermediate PRS should be intensively intervened in an ideal lifestyle, while only 29 people with high PRS should be intensively intervened. The Eastern Asian PRS could also refine the risk stratification defined by the current guideline-recommended CDRS. These findings provide a basis for the application of the PRS in the early identification of high-risk individuals and a precise intervention of T2D.

GWAS studies have so far reported over 700 novel T2D risk loci [12]. However, with the increased sample size of participants, the effect value of newly discovered risk variants decreased. In our current study, we selected and genotyped the loci mostly reported in the first and second waves of GWAS, which have relatively high effects. The PRS based on the 46 SNPs identified in Eastern Asians can be used to predict the risk of T2D in Chinese, while the PRS based on 50 SNPs identified in Europeans did not reach significance. The result is consistent with previous studies showing that the PRS derived from genetic variations identified by GWAS in Europe can lead to differences in the accuracy of risk prediction among different races [57,58]. It is suggested that the PRS has ethnic specificity in clinical practice, and it is necessary to construct a PRS model suitable for the population to identify individuals at high genetic risk.

One of the key public health needs in China is to identify individuals at high risk of T2D for precise intervention. The PRS has been successfully developed to measure genetic risk for NCDs, including coronary artery disease, stroke, and cancer [15,16,17]. Our findings provided evidence that the Eastern Asian PRS could be regarded as a robust predictor for T2D, which when incorporated with traditional clinical risk predictors, might be used for the early identification of high-risk individuals for healthy lifestyle interventions, and to improve risk classification for optimizing blood glucose management strategies.

Previous studies have confirmed that healthy lifestyle was inversely associated with the risk of T2D [6]. Consistent with previous studies, our results indicated that intermediate and poor lifestyles significantly increase the risk of T2D incidence. Taking genetic data and lifestyle together, our study showed that an ideal lifestyle could significantly reduce T2D risk within the intermediate- and high-genetic-risk groups, whereas the effect was not found in the low-genetic-risk group. The effect of PRS in the population at high genetic risk could be offset by adhering to a healthy lifestyle, leading to a reduction in their overall risk of T2D by nearly 60%. Using the PRS to identify high-genetic-risk individuals for intervention will save costs and improve the effectiveness of the intervention. An effective behavioral intervention before the formation of a poor lifestyle for individuals at high genetic risk is probably an effective precaution for T2D prevention.

Interestingly, among participants with low clinical risk according to the CDRS recommended by the Chinese Guidelines for Diabetes Prevention and Treatment (2020 edition), the 10-year absolute risk for those with the high genetic risk would exceed the level of the high-clinical-risk group with low genetic risk. Recently, evidence showed that current models of clinical risk assessment did not identify individuals at high genetic risk of NCDs well, and the PRS was considered a risk-enhancing factor to up-classify risk in clinical applications [59]. Our study suggested that individuals with low clinical risk but high genetic risk should also undergo an intensive intervention (such as blood glucose monitoring, Oral Glucose Tolerance Test detection, and adopting an ideal lifestyle), as recommended for high-clinical-risk groups.

There are several strengths in our study, including a prospective cohort design with up to 14 years of follow-up, a comparison of the European PRS and Eastern Asian PRS in predicting T2D risk, and standardized protocols to assess heritable, clinical factors and lifestyle effects. Nevertheless, several limitations should also be stressed. First, due to the ethnic specificity of the PRS, caution must be taken when generalizing our results to other ethnic populations. Second, some lifestyle factors, such as physical activity and diet, were self-reported, which might result in the misclassification of lifestyle risk levels. Third, lifestyles were only assessed at baseline, and the risk estimate was conducted without the consideration of behavior changes during the follow-up period.

We constructed and validated an Eastern Asian PRS in the Wuxi NCDs cohort, which can be used to predict the future risk of T2D in Chinese Hans. The detrimental effects of genetic risk on T2D could be attenuated by adopting an ideal lifestyle. Among those with high genetic risk identified by the PRS, the risk of T2D in those with an ideal lifestyle could be reduced by more than 50% compared with those with a poor lifestyle. The PRS could also refine the risk classification defined by traditional clinical risk factors. These results indicate that the Eastern Asian PRS could be effectively used for T2D risk prediction and potentially applied in identifying high-risk individuals for precise prevention of T2D in the Chinese Hans population.

## Figures and Tables

**Table 1:** The association between characteristics at baseline and type 2 diabetes risk.

Characteristics at Baseline	No. (%) in Cohort	No. (%) in T2D Incidence	Log-Rank P	HR (95% CI)
Age >52 years ^1^	2390 (47.61)	309 (70.23)	<2.00 × 10^−16^	2.93 (2.39–3.60)
Female	3022 (60.15)	261 (59.32)	0.535	0.94 (0.78–1.14)
Systolic blood pressure >120 mmHg ^2^	2014 (40.14)	238 (54.21)	4.25 × 10^−11^	1.88 (1.56–2.27)
Diastolic blood pressure >80 mmHg ^2^	1291 (25.74)	156 (35.54)	4.48 × 10^−7^	1.65 (1.36–2.01)
Fasting blood glucose >4.5 mmol/L ^2^	2169 (43.40)	324 (73.80)	<2.00 × 10^−16^	3.97 (3.21–4.91)
Total cholesterol ≥5.2 mmol/L ^2^	1300 (26.02)	149 (33.94)	6.46 × 10^−5^	1.50 (1.23–1.82)
Triglycerides ≥1.7 mmol/L ^2^	2128 (42.61)	284 (64.84)	<2.00 × 10^−16^	2.56 (2.10–3.11)
High density lipoprotein cholesterol <1.0 mmol/L ^2^	502 (10.05)	77 (17.58)	5.00 × 10^−8^	1.98 (1.55–2.54)
Family history of diabetes	483 (9.72)	54 (12.27)	0.088	1.28 (0.96–1.74)
Overweight/obesity	1810 (36.10)	256 (58.32)	<2.00 × 10^−16^	2.60 (2.15–3.15)
Central adiposity	2624 (52.34)	317 (72.37)	<2.00 × 10^−16^	2.50 (2.03–3.08)
Smoking	1047 (20.94)	104 (23.64)	0.121	1.19 (0.96–1.48)
Drinking	462 (9.24)	47 (10.73)	0.209	1.21 (0.90–1.64)
No physical exercise	3236 (65.01)	294 (67.12)	0.386	1.10 (0.91–1.35)
Unhealthy diet	4004 (80.06)	364 (82.73)	0.146	1.20 (0.94–1.54)
Intermediate lifestyle ^3^	1169 (23.76)	133 (30.65)	3.11 × 10^−4^	1.58 (1.23–2.03)
Poor lifestyle ^3^	1262 (25.65)	177 (40.78)	3.49 × 10^−7^	1.92 (1.49–2.46)

^1^ Categorized by median value of age and fasting blood glucose at baseline. ^2^ Category criteria based on the guidelines for prevention and treatment of hypertension in China and the guidelines for prevention and treatment of dyslipidemia in China. Ideal blood pressure (systolic blood pressure ≤120 mmHg and diastolic blood pressure ≤80 mmHg), total cholesterol (<5.2 mmol/L), triglyceride (<1.7 mmol/L), and high-density lipoprotein cholesterol (≥1.0 mmol/L) are regarded as reference group. ^3^ Overall lifestyle was categorized as poor (0–2 healthy lifestyle factors), intermediate (3 healthy lifestyle factors), and ideal (4–6 healthy lifestyle factors). Ideal lifestyle was regarded as reference group.

**Table 2:** Risk of incident type 2 diabetes according to different lifestyles and genetic risk.

	HR (95% CI)	*P*	*P*_trend_	Absolute Risk over 10 Years (%)	Absolute Risk Reduction over 10 Years (%)	Number of Participants Who Need to Adhere to Healthy Lifestyle
**Low genetic risk**			0.112			
Poor lifestyle	1	-		3.52 (1.30–5.74)	1 (ref)	
Intermediate lifestyle	0.55 (0.24–1.24)	0.151		1.93 (0.57–3.30)	1.58 (−1.07–3.42)	63
Ideal lifestyle	0.52 (0.24–1.14)	0.103		1.83 (0.72–2.95)	1.68 (−0.88–3.64)	60
**Intermediate genetic risk**			9.96 × 10^−5^			
Poor lifestyle	1	-		4.73 (3.28–6.19)	1 (ref)	
Intermediate lifestyle	0.75 (0.52–1.09)	0.130		3.55 (2.41–4.70)	1.18 (−0.47–2.75)	85
Ideal lifestyle	0.45 (0.30–0.68)	1.05 × 10^−4^		2.15 (1.45–2.85)	2.58 (0.96–4.10)	39
**High genetic risk**			0.014			
Poor lifestyle	1	-		6.77 (3.74–9.80)	1 (ref)	
Intermediate lifestyle	0.60 (0.34–1.04)	0.070		4.04 (2.02–6.06)	2.73 (−0.76–6.06)	37
Ideal lifestyle	0.48 (0.28–0.85)	0.012		3.28 (1.83–4.74)	3.49 (0.05–6.80)	29

Polygenic risk categories: low (bottom quintile), intermediate (quintile 2–4), and high (top quintile) genetic risk according to quintiles of Eastern Asian PRS. Healthy lifestyle categories: ideal (4–6 healthy lifestyle factors), intermediate (3 healthy lifestyle factors), and poor lifestyle (0–2 healthy lifestyle factors).

## Data Availability

The data presented in this study are available on request from the corresponding author. The data are not publicly available due to privacy reasons.

## Supplementary Materials

The following supporting information can be downloaded at: https://www.mdpi.com/article/10.3390/nu15092144/s1, Figure S1: Flow chart of selection process of study participants and SNPs; Figure S2: The HRs of incident T2D in different genetic and clinical risk score categories in Wuxi NCDs cohort; Table S1: Basic information of selected 80 SNPs to build PRS.
