# Supplementary material for: Polygenic Risk Score, Lifestyles, and Type 2 Diabetes Risk: A Prospective Chinese Cohort Study
Source: Nutrients. 2023 Apr 29;15(9):2144. doi: 10.3390/nu15092144 (PMC10181480; doi:10.3390/nu15092144)
Supplement: Supplementary file 1 [file nutrients-15-02144-s001.zip › nutrients-2374895-supplementary.pdf]

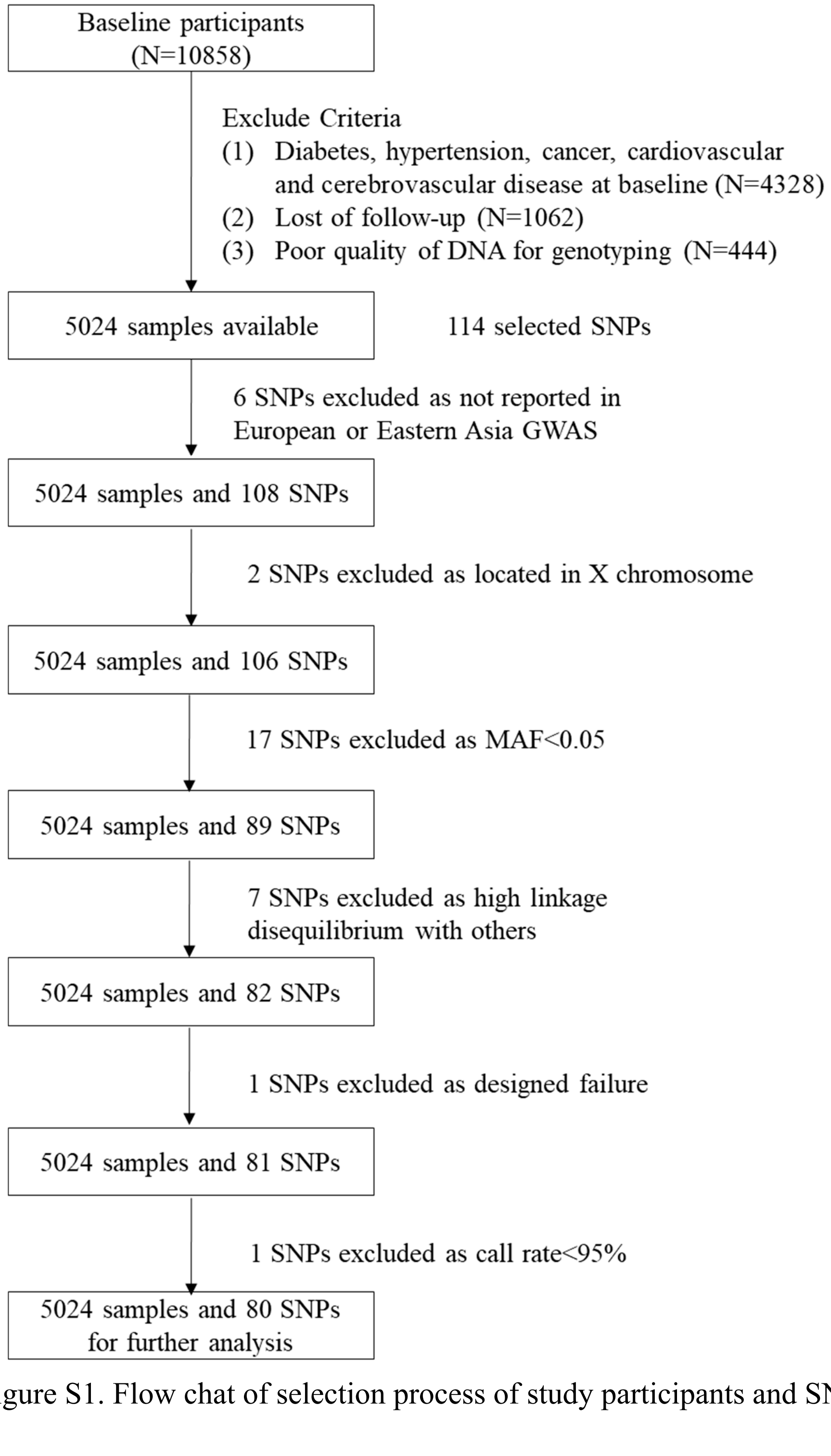

Figure S1. Flow chat of selection process of study participants and SNPs

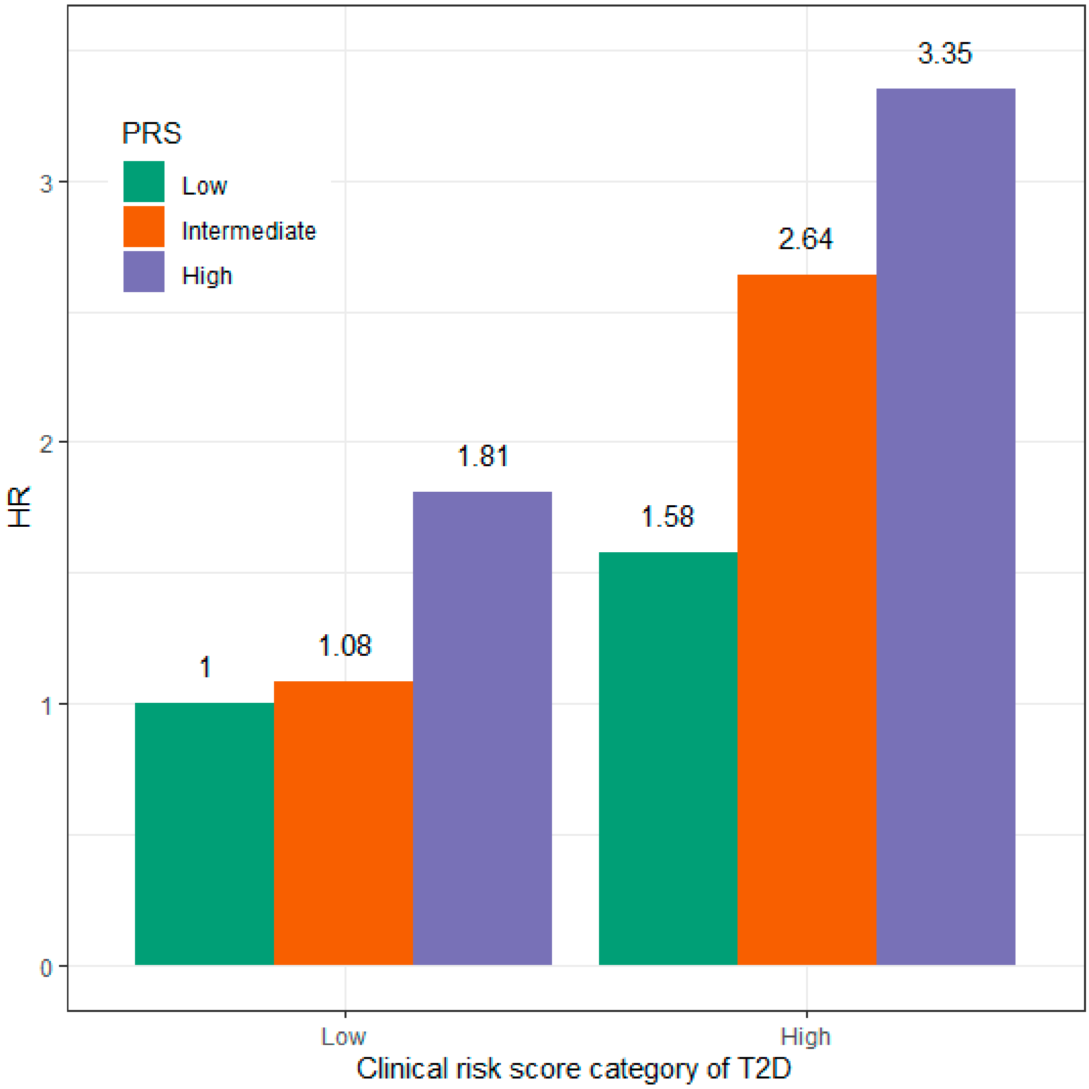

Figure S2. The HRs of incident T2D in different genetic and clinical risk score categories in Wuxi NCDs cohort

Polygenic risk categories: low (bottom quintile), intermediate (quintile 2-4), high (top quintile) genetic risk according to quintiles of Eastern Asian-derived PRS. Clinical risk score categories: low (Chinese diabetes risk score≤25); high (Chinese diabetes risk score>25), recommended by Chinese Guidelines for Diabetes Prevention and Treatment (2020 edition).

Table S1. Basic information of selected 80 SNPs to build PRS

Chr	SNP rsID	Mapped Gene	Effect Alleles	EAF	Beta	PMID	Ancestry
1	rs12742393	NOS1AP	C	0.24	0.16	19937226	Eastern Asian
1	rs17106184	FAF1	G	0.89	0.10	24509480	European
2	rs243021	MIR4432HG	A	0.66	0.08	20581827	European
2	rs243088	MIR4432HG	T	0.67	0.07	22885922	European
2	rs3923113	GRB14	A	0.86	0.10	22885922	European
2	rs7578326	IRS1	A	0.86	0.10	20581827	European
3	rs1470579	IGF2BP2	C	-	0.13	20581827	European
			C	0.27	0.17	26818947	Eastern Asian
3	rs4402960	IGF2BP2	T	0.33	0.12	22885922	European
			T	0.26	0.13	19401414	Eastern Asian
3	rs6780569	UBE2E2-AS1	G	0.79	0.16	23945395	Eastern Asian
3	rs831571	PSMD6	C	0.63	0.09	22158537	Eastern Asian
3	rs1801282	PPARG	C	0.94	0.08	29632382	European
3	rs4607103	ADAMTS9-AS2	C	0.63	0.09	18372903	European
4	rs6815464	MAEA	G	0.43	0.11	23945395	Eastern Asian
4	rs6813195	TMEM154	C	0.54	0.08	24509480	European
5	rs459193	C5orf67	G	0.70	0.08	22885922	European
			G	0.54	0.07	32514122	Eastern Asian
6	rs1535500	KCNK16	T	0.59	0.12	24509480	European
			T	0.45	0.08	22158537	Eastern Asian
6	rs6918311	RPL35AP3	A	0.53	0.06	28566273	European
			G	0.51	0.08	32514122	Eastern Asian
6	rs9470794	ZFAND3	C	0.30	0.11	22158537	Eastern Asian
6	rs2206734	CDKAL1	A	0.40	0.22	32794382	Eastern Asian
6	rs9472138	VEGFA	T	0.11	0.17	22437209	Eastern Asian
6	rs3130501	POU5F1-TCF19	G	0.70	0.07	24509480	European

6	rs6931514	CDKAL1	G	0.50	0.22	18372903	European
6	rs9271774	HLA-DQA1	C	0.62	0.09	28566273	European
6	rs9505118	SSR1	A	0.57	0.06	24509480	European
7	rs17168486	DGKB	T	0.18	0.07	30297969	European
			T	0.45	0.07	32499647	Eastern Asian
7	rs6467136	GCC1	G	0.76	0.10	22158537	Eastern Asian
7	rs10229583	PAX4	G	0.82	0.13	23532257	Eastern Asian
7	rs864745	JAZF1	T	0.77	0.10	18372903	European
7	rs972283	KLF14	G	0.83	0.07	20581827	European
8	rs13266634	SLC30A8	C	0.68	0.09	29632382	European
			C	0.57	0.11	26818947	Eastern Asian
8	rs896854	TP53INP1	T	0.33	0.06	20581827	European
9	rs10811661	CDKN2B-AS1	T	-	0.19	26961502	European
			T	0.52	0.21	23945395	Eastern Asian
9	rs2383208	CDKN2B-AS1	A	0.55	0.29	19401414	Eastern Asian
9	rs10814916	GLIS3	C	0.45	0.10	22961080	Eastern Asian
9	rs17584499	PTPRD	T	0.13	0.45	20174558	Eastern Asian
9	rs635634	ABO	T	0.83	0.08	28566273	European
10	rs1111875	HHEX	C	0.58	0.10	22885922	European
			C	0.27	0.13	23945395	Eastern Asian
10	rs11257655	CDC123	T	0.22	0.09	30297969	European
			T	0.57	0.11	26818947	Eastern Asian
10	rs7903146	TCF7L2	T	0.30	0.31	30297969	European
			T	0.06	0.39	23945395	Eastern Asian
10	rs10886471	GRK5	C	0.79	0.11	22961080	Eastern Asian
10	rs10906115	CDC123	A	0.63	0.12	20862305	Eastern Asian
10	rs5015480	HHEX	C	0.17	0.16	20862305	Eastern Asian
10	rs7923837	HHEX	G	0.20	0.29	22506066	Eastern Asian
10	rs12571751	ZMIZ1	A	0.58	0.08	22885922	European

10	rs12779790	CDC123	G	0.18	0.10	18372903	European
10	rs2292626	PLEKHA1	C	0.36	0.07	28566273	European
11	rs2237895	KCNQ1	C	0.31	0.22	32794382	Eastern Asian
11	rs2237897	KCNQ1	C	0.95	0.21	30297969	European
			C	0.63	0.25	32499647	Eastern Asian
11	rs5219	KCNJ11	T	0.47	0.13	17463246	European
			T	0.39	0.04	30718926	Eastern Asian
11	rs10751301	TENM4	C	0.21	0.10	20862305	Eastern Asian
11	rs1552224	ARAP1	C	0.91	0.25	32514122	Eastern Asian
11	rs231359	KCNQ1	A	0.80	0.29	20174558	Eastern Asian
11	rs1061810	HSD17B12	A	0.21	0.05	30297969	European
11	rs111669836	KCNK7	A	0.12	0.06	28566273	European
11	rs1387153	MTNR1B	T	0.42	0.09	20581827	European
11	rs163184	KCNQ1	G	0.43	0.09	22885922	European
11	rs231361	KCNQ1	A	0.80	0.08	30297969	European
12	rs10842994	PTHLH	C	0.82	0.08	30297969	European
12	rs4275659	MPHOSPH9	C	0.66	0.06	24509480	European
12	rs7961581	TSPAN8	C	0.22	0.09	18372903	European
13	rs1359790	SPRY2	G	0.82	0.09	30297969	European
15	rs2028299	AP3S2	C	0.29	0.04	24509480	European
			C	0.20	0.12	23945395	Eastern Asian
15	rs7172432	C2CD4B	A	0.61	0.10	20818381	Eastern Asian
15	rs1436955	C2CD4B	C	0.77	0.12	20862305	Eastern Asian
15	rs7403531	RASGRP1	T	0.36	0.10	22961080	Eastern Asian
15	rs7177055	HMG20A	A	0.67	0.08	22885922	European
15	rs7178572	HMG20A	G	0.36	0.08	24509480	European
16	rs16955379	CMIP	C	0.74	0.08	22158537	Eastern Asian
16	rs2925979	CMIP	T	0.30	0.05	30297969	European

			T	0.41	0.04	32499647	Eastern Asian
16	rs17797882	WWOX	T	0.21	0.08	22158537	Eastern Asian
16	rs7192960	MAF-WWOX	C	0.72	0.19	20174558	Eastern Asian
16	rs8050136	FTO	A	0.11	0.16	24053193	Eastern Asian
16	rs7202877	CTRB2	T	0.80	0.11	22885922	European
17	rs4430796	HNF1B	G	-	0.13	20581827	European
			G	0.29	0.10	23945395	Eastern Asian
17	rs391300	SRR	G	0.70	0.25	20174558	Eastern Asian
18	rs12970134	RNU4-17P	A	0.19	0.08	22885922	European
19	rs3786897	PEPD	A	0.52	0.10	22158537	Eastern Asian
19	rs8108269	GIPR	G	0.53	0.07	22885922	European
20	rs6017317	FITM2	G	0.43	0.09	22158537	Eastern Asian
20	rs4812829	HNF4A	A	0.46	0.07	24509480	European

EAF: Effect allele frequency
